# 2697. Fungal Infection Patterns in Hematopoietic Stem Cell Transplant Patients of a Tertiary Care Indian Cancer Hospital

**DOI:** 10.1093/ofid/ofad500.2308

**Published:** 2023-11-27

**Authors:** Soumyadip Chatterji, Arijit Nag, Jeevan Kumar, Debranjani Chattopadhyay, Dibakar Podder, Reena Nair, Parijat Das, Sanjay Bhattacharya, Gaurav Goel, Mammen Chandy

**Affiliations:** Tata medical center kolkata, Kolkata, West Bengal, India; Tata medical center kolkata, Kolkata, West Bengal, India; Tata medical center kolkata, Kolkata, West Bengal, India; Tata medical center kolkata, Kolkata, West Bengal, India; Tata medical center kolkata, Kolkata, West Bengal, India; Tata medical center kolkata, Kolkata, West Bengal, India; Tata medical center kolkata, Kolkata, West Bengal, India; Tata medical center kolkata, Kolkata, West Bengal, India; Tata medical center kolkata, Kolkata, West Bengal, India; Tata medical center kolkata, Kolkata, West Bengal, India

## Abstract

**Background:**

Fungal infections are a common and serious complication in patients undergoing Hematopoietic Stem Cell Transplantation (HSCT). Despite the use of prophylactic antifungal therapy, breakthrough infections can still occur and lead to significant morbidity and mortality. The emergence of antifungal resistance has further complicated the management of these infections. Therefore, there is a pressing need to understand breakthrough fungal infection patterns in HSCT patients.

**Methods:**

In this record-based observational descriptive study, we attempted to understand the fungal infection patterns in HSCT patients in our hospital. Cases were analysed from 2012 to 2021. Diagnosis of invasive fungal infection (IFI) was done as per EORTC MSG criteria. For the patients who were on prophylactic antifungal agents, date of sample collection was tallied with the period of medication to determine eligibility for breakthrough invasive fungal infection (BIFI).

**Results:**

Out of 563 recruited cases, 16 fungal infections were confirmed. Responsible pathogens were identified as Aspergillus sp. (11 by Galactomannan antigen test, 1 by DNA PCR from blood), Candida sp. (1 by PCR, 1 by blood culture, 1 by mannan antigen) and Fusarium sp. (1 by blood culture), see Fig.1.

Number of patients receiving (treatment/prophylaxis) each type of antifungal agent is shown in Fig. 2. Antifungal prophylaxis was administered in 544 patients (Fluconazole n=219, 40.3%; Voriconazole n=2, 0.4%; Posaconazole n=310, 57%; Amphotericin B n=13, 2.4%; see Fig. 3). Further analysis yielded 14 cases of BIFI (10 on Posaconazole and 4 on Fluconazole prophylaxis).

**Figure:**
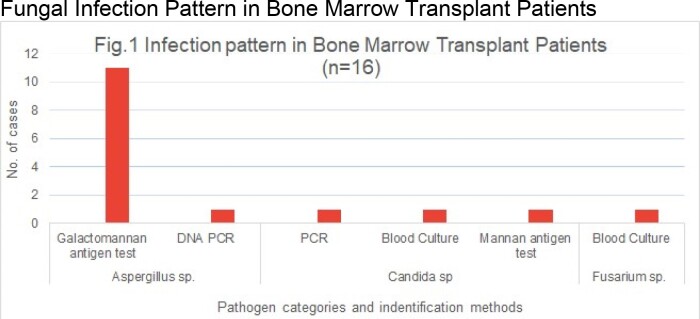

Number of patients receiving each type of antifungal

**Figure:**
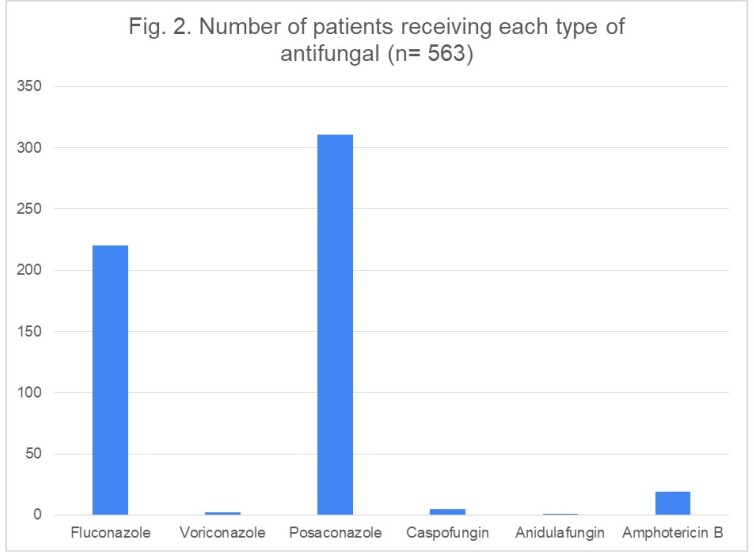

Percentage of patients on prophylactic antifungal drug

**Figure:**
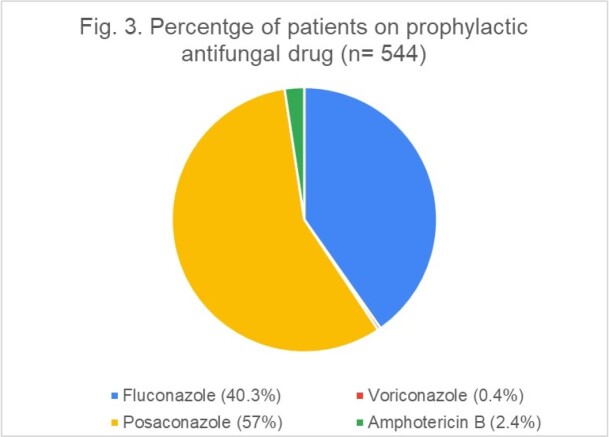

**Conclusion:**

These results thoroughly describe the pattern of fungal infection in HSCT patients of the hospital for the first time.

**Disclosures:**

**All Authors**: No reported disclosures

